# Insight Into TLR4-Mediated Immunomodulation in Normal Pregnancy and Related Disorders

**DOI:** 10.3389/fimmu.2020.00807

**Published:** 2020-05-19

**Authors:** Priyanka Firmal, Vibhuti Kumar Shah, Samit Chattopadhyay

**Affiliations:** ^1^National Centre for Cell Science, S. P. Pune University Campus, Pune, India; ^2^Department of Biological Sciences, BITS Pilani, K. K. Birla Goa Campus, Goa, India; ^3^Indian Institute of Chemical Biology, Kolkata, India

**Keywords:** TLR4, preterm birth, pregnancy, pro-inflammatory, innate immunity

## Abstract

Unlike organ transplants where an immunosuppressive environment is required, a successful pregnancy involves an extremely robust, dynamic, and responsive maternal immune system to maintain the development of the fetus. A specific set of hormones and cytokines are associated with a particular stage of pregnancy. Any disturbance that alters this fine balance could compromise the development and function of the placenta. Although there are numerous underlying causes of pregnancy-related complications, untimely activation of Toll-like receptors (TLR), primarily TLR4, by intrauterine microbes poses the greatest risk. TLR4 is an important Pattern Recognition Receptor (PRR), which activates both innate and adaptive immune cells. TLR4 activation by LPS or DAMPs leads to the production of pro-inflammatory cytokines via the MyD88 dependent or independent pathway. Immune cells modulate the materno–fetal interface by TLR4-mediated cytokine production, which changes at different stages of pregnancy. In most pregnancy disorders, such as PTB, PE, or placental malaria, the TLR4 expression is upregulated in immune cells or in maternal derived cells, leading to the aberrant production of pro-inflammatory cytokines at the materno–fetal interface. Lack of functional TLR4 in mice has reduced the pro-inflammatory responses, leading to an improved pregnancy, which further strengthens the fact that abnormal TLR4 activation creates a hostile environment for the developing fetus. A recent study proposed that endothelial and perivascular stromal cells should interact with each other in order to maintain a homeostatic balance during TLR4-mediated inflammation. It has been reported that depleting immune cells or supplying anti-inflammatory cytokines can prevent PTB, PE, or fetal death. Blocking TLR4 signaling or its downstream molecule by inhibitors or antagonists has proven to improve pregnancy-related complications to some extent in clinical and animal models. To date, there has been a lack of knowledge regarding whether TLR4 accessories such as CD14 and MD-2 are important in pregnancy and whether these accessory molecules could be promising drug targets for combinatorial treatment of various pregnancy disorders. This review mainly focuses on the activation of TLR4 during pregnancy, its immunomodulatory functions, and the upcoming advancement in this field regarding the improvement of pregnancy-related issues by various therapeutic approaches.

## Introduction

Pregnancy is an immunologically unique state owing to the fact that it requires the maternal immune system to be highly active so as to fight the upcoming intrauterine microbial challenges, but it is also simultaneously required to be immunosuppressed to maintain the semi-allogenic fetal development (1–3). A fine interplay between both phases ensures a healthy pregnancy. There are numerous reports that have suggested that any dysregulation in the immune status at the materno–fetal interface due to infections are the main cause of preterm delivery, preeclampsia, gestational diabetes, miscarriage, placental malaria, and other pregnancy-related disorders (4–7). There are multiple routes through which the infections can gain access to the placenta, maternal endometrium, and amniotic fluid; ascending through the genital tract and colonizing uterine cavity is the most preferred of all (8). Many of these microbial components act as a ligand for the pattern recognition receptors (PRRs). Pattern recognition receptors are an important element of the innate immune system since they act as a first line of defense against invading pathogens. Recognition of microorganism-originated pathogen-associated molecular patterns (PAMPs) or host-derived damage-associated molecular patterns (DAMPs) relays the signaling cascade, leading to an increase in the expression of cytokines, chemokines, and interferons (7, 9). The Toll-like receptor (TLR) family is one of the important subgroups of PRRs, and it acts as a bridge between innate and adaptive immunity. Expression of TLRs is not restricted to immune cells, but they are also present on variety of cell types, including fibroblasts, endothelial cells, and epithelial cells, and also on placental tissue (10, 11). Each TLR recognizes a specific microbial product and activates a defined signaling pathway leading to distinct immunological response. There are numerous studies that have reported that administration of a TLR4-specific ligand, lipopolysaccharide (LPS), stimulated the generation of pro-inflammatory cytokines and prostaglandins in gestational tissues that leads to preterm labor (12–14). This review emphasizes the role of TLR4 signaling in normal pregnancy and its dysregulation leading to adverse outcomes. We will also summarize promising therapeutic strategies that focus on targeting the TLR4 signaling pathway for the management of pregnancy-related disorders.

## Toll-Like Receptors

The *Toll* gene was first discovered in Drosophila, where it plays a critical role in defining the dorso–ventral axis during embryonic development (15). A few key findings revealed that the Toll protein is involved in imparting an immune response against fungi and bacteria in adult fly (16, 17). Later, receptors similar to Toll were identified in humans, and the first one was mapped on chromosome 4 (18, 19). During that time, TLRs were believed to be important in the development process. Subsequently, however, human homologs of Drosophila Toll, TLRs, were also reported to be involved in activating innate and adaptive immune responses in vertebrates. There are a total of 10 homologs of TLR (TLR1-TLR10) that are known to be expressed by humans and that can specifically detect different surface and intracellular pathogen products.

Toll-like receptors (TLRs) comprise of an extracellular domain, including leucine-rich repeats and a Toll/interleukin-1 receptor (TIR) domain at the cytoplasmic end. Following ligand recognition, TLRs relay the signaling either via the intracellular signaling adapter protein, the myeloid differentiation factor 88 (MyD88)-dependent pathway, or the MyD88-independent pathway, which is also known as the TLR-mediated TIR-domain-containing adapter-inducing interferon-β (TRIF)-dependent pathway. The MyD88-dependent pathway leads to the activation of early phase nuclear factor-κB (NF-κB), resulting in the production of pro-inflammatory cytokines, including IL-1β, IL-6, IL-12, and TNF-α. The TRIF-dependent pathway generates Type I IFNs (IFNα /β) through interferon regulatory factor (IRF-3) and via activation of late-phase NF-κB (20, 21).

Proper release of these cytokines by the activated leukocytes or uterine epithelial cells plays a key role in attaining a successful pregnancy by facilitating the fetus implantation. But there is increasing evidence to suggest that uncontrolled activation of TLRs—either on leukocytes or uterine epithelial and stromal cells, specifically TLR4—at the materno-uterine junction is associated with pregnancy-related problems (22–25).

### Extracellular Receptor Complex

TLR4 in itself is unable to recognize LPS, and it therefore requires numerous other proteins for ligand recognition. The LPS-binding protein (LBP) is one such soluble plasma protein that first interacts with LPS and then transfers it to a cluster of differentiation 14 (either sCD14 or membrane bound). CD14 is a GPI-linked protein that is also one of the PRRs that can bind to the LPS-LBP complex; finally, it also chaperones the LPS molecule to MD-2/TLR4 signaling complex. Myeloid differentiation 2 (MD-2) is an adapter protein that directly recognizes and binds to the conserved lipid A moiety of LPS (26, 27). The intracellular signaling is triggered only when MD-2 interacts non-covalently on the extracellular domain of TLR4 to forms a heterodimeric complex (LPS.MD-2.TLR4)_2_ (28).

### TLR4 Signal Transduction

**Figure 1:**
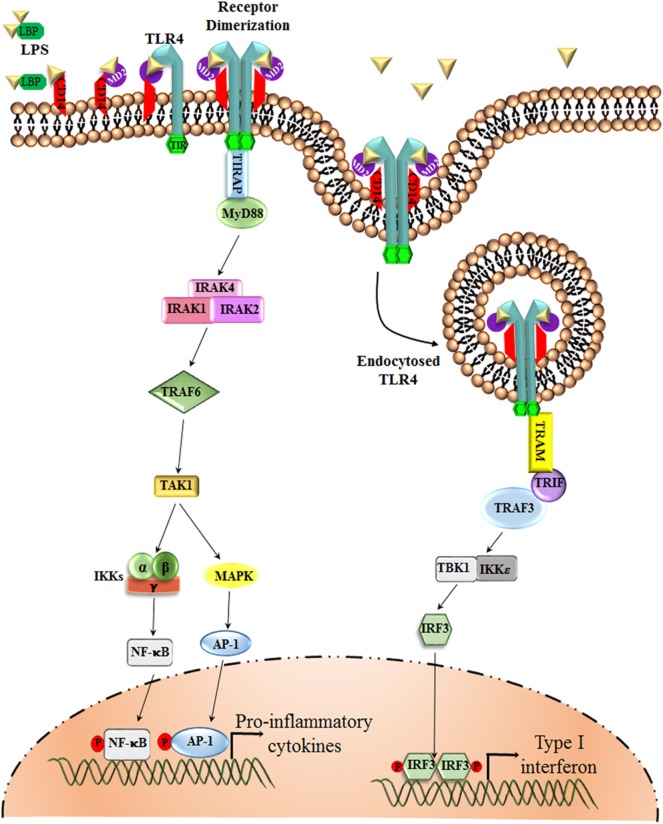
TLR4 Signaling pathway. The LPS Binding Protein (LBP) binds to LPS and transfers it to CD14 or MD-2, which are the accessory proteins involved in the ligand recognition, dimerization, and endocytosis of TLR4. TLR4, upon dimerization, can signal via two separate pathways, the MyD88-dependent and the MyD88-independent pathway. The MyD88-dependent pathway involves the activation of IRAKs and TRAF6, which results in the phosphorylation of transcription factors, such as NF-κB and AP-1. These transcription factors upon phosphorylation translocate to the nucleus and are involved in triggering the transcription of pro-inflammatory cytokine genes. The MyD88-independent pathway, or the TRIF-dependent pathway, however, involves TRAF3 for the activation of transcription factor IRF-3, which favors the production of Type I interferons, such as IFN α, β.

TLR4, the first identified human Toll-like receptor, is the only TLR that can signal via an MyD88-dependent as well as an MyD88-independent manner. It acts as a specific receptor for gram-negative bacterial lipopolysaccharide (LPS) and can also bind DAMPs, such as hyaluronic acid and β-defensin 2, fibrinogen, and heat shock proteins hsp60 and hsp70 (29, 30). The binding of the ligand to the receptor triggers the intracellular signaling pathway. Each TLR shares a similar cytoplasmic signaling domain, which is similar to the IL-1 receptor, the TIR domain. Numerous adaptor molecules that have a TIR domain, such as MyD88, TRIF, TIR domain-containing adaptor protein/MyD88 adapter-like protein (TIRAP/Mal), and TRIF-related adaptor molecule (TRAM), interact with the TIR domain of TLR4 and thus relay the downstream signal. Among all the TLRs, TLR3 is the only one that does not signal via the MyD88-dependent pathway. Furthermore, only TLR4 utilizes all of the four adaptor molecules, namely, MyD88, TIRAP, TRIF, and TRAM, for signal transduction (9, 31) (Figure 1).

There are numerous reports that emphasizes the role of immune activation in the intestinal and respiratory tract, and a wealth of knowledge is currently focused on uterine epithelial cells of the female reproductive tract (FRT) being an essential immunological site (32–36). Several studies have shown that TLRs are expressed all through the pregnancy at different locations in the FRT (37, 38). Expression of PRRs on the epithelial and stromal cells in the uterus helps in the recognition and timely response toward vaginal infections. Conversely, the uncontrolled activation of innate immune system may also result in poor pregnancy outcomes.

#### MyD88-Dependent Signaling

After the dimerization of TLR4 on ligand binding, MyD88 is recruited, and it interacts via its TIR domain to the cytoplasmic region of TLR4 through a homophilic interaction. Several other accessory molecules are also employed, including various IL-1 receptor-associated kinases (IRAKs), TRAFs, and mitogen-activated protein kinases (MAPKs). Next, NF-κB is activated and translocated to the nucleus via initiating the degradation of its inhibitory protein Iκ-Bα by inhibitory kappa B kinase (IKK). Activating protein-1 (AP-1) is one of the transcription factors that is activated by MAPKs (31). This pathway ultimately leads to the production of several pro-inflammatory cytokines and chemokines.

#### MyD88-Independent Signaling

TLR4/TRIF dependent signaling is only initiated after the receptor complex is internalized into the endosomes. Only TLR3 and TLR4 utilizes this pathway, involving the participation of TRIF and IRF-3 and resulting in the production of type I interferons (IFN) along with pro-inflammatory cytokines. They have the capability to stimulate IFN-β and Interferon-inducible genes in *MYD88* null cells owing to the fact that both the pathways need different accessory proteins to function (9). IRF-3 and IRF-7, upon phosphorylation, dimerize and translocate to the nucleus where they bind to the Interferon-Stimulated Response Elements (ISREs), giving rise to the expression of interferon-inducible genes. IRF-3 and IRF-7 are crucial among the IRF family, as Type I interferon production is severely hampered in *IRF-7* null mice and was completely abolished in *IRF-3* and *IRF-7* null cells (39). TLR4-induced type I IFN induction was highly compromised in *IRF-3* null mice emphasizing the importance of IRF-3 and IRF-7 in TLR signaling pathway (40). Interestingly, there are reports that have highlighted that CD14 plays a major role in supporting the internalization of (LPS.MD-2.TLR4)_2_ receptor into the endosomes (41).

### TLR4 Expression and Signaling at the Materno–Fetal Interface

**Figure 2:**
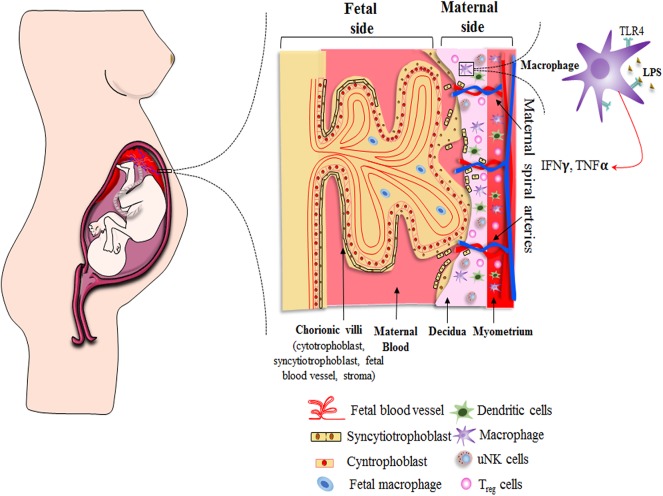
Schematic showing distribution of immune cells across materno–fetal interface during early pregnancy. Macrophages, Treg cells, and dendritic cells are present in the myometrium as well as decidua, while uterine natural killer (uNK) cells are restricted to decidua. The effective crosstalk between various immune cells and extravillous trophoblast cells creates an immunosuppressive environment and helps in the formation of various pregnancy-related tissues, both of which are essential for a successful pregnancy. Extravillous cytotrophoblasts enter the decidua to reach out to maternal spiral arteries for obtaining required nutrient to support developing embryo. Other nutrients, gas, and waste exchange happens via placental villus, which interacts with the maternal blood directly. The villus has double layer of cells consisting of syncytiotrophoblasts and cytotrophoblasts. It encloses the fetal blood vessels along with fibroblasts and fetal macrophages (Hofbauer cells). Immune cells as well as placental cells protect the fetus by expressing PRRs, such as Toll-like receptors, on their surface throughout the pregnancy.

Histological and functional changes of different parts of the female reproductive tract involving the perimetrium, myometrium, endometrium, cervix, and vagina take place throughout normal pregnancy. Several pregnancy-related tissues are also formed, including the amnion, chorion, and placenta, to support the development of the fetus. Any dysregulation in the usual scenario results in adverse pregnancy outcomes. Hence, in the current review, we have focused on the investigations that have been carried out to look into the function and expression profile of TLR4 during the course of pregnancy, exploring specific materno–fetal tissues of the female reproductive tract that have a close relationship with the developing embryo (Figure 2).

### Placenta

The developing embryo is protected from the surrounding environment effectively by the placenta. Numerous PRRs are contemplated to take part in this interface, including Nod-like receptors (NLRs) and TLRs (42). All TLRs are found to be present in the normal-term placental tissue at the mRNA level, but only TLR2 and TLR4 are completely characterized at the protein level. The expression of these receptors is not continuous throughout the pregnancy but follows a definitive trend. In the first trimester placental tissues, trophoblast cells exhibit enhanced expression of TLR2 and TLR4. The villous cytotrophoblast along with extravillous trophoblast expresses TLR4 in first trimester trophoblast. The outer syncytiotrophoblast cells that directly interact with the maternal blood are found to lack TLR4 expression (43). Therefore, a pathogen can get access to the placenta by crossing the syncytiotrophoblast cell layer that is lacking in TLR4 and pose a threat to the inner placental compartments. The entrance of a pathogen into the trophoblast cell expressing TLR4, however, results in excessive chemokine secretion, which leads to enhanced chemotaxis of a monocyte and neutrophil to the site of infection (44).

The differential expression of TLRs persists till the end of the second trimester. There are various reports that suggest that TLR4 is expressed during the second and third trimester in human placentas obtained from normal and preterm pregnancies. The expression of TLR4 has also been found in the syncytiotrophoblast layer by the third trimester. These studies have signified that placental cells can effectively counter the intrauterine infections (45, 46). A recent study has now focused on the temporal changes of TLRs expression taking place throughout gestation, which can help in devising an effective clinical diagnostic marker by observing the TLR pattern shifts at the materno–fetal interface during pregnancy (11). Another study elucidated the mechanism that regulates IFN-β expression in the trophoblast through a negative feedback loop to ensure an effective response against invading pathogens (47).

### Fetal Membranes

Chorioamnionitis is characterized by the inflammatory response generated in the amnion and chorion membranes by the invading pathogenic microbes, resulting in preterm labor (48). Fetal membrane infections are known to trigger pro-inflammatory cytokines, in particular IL-6, TNF-α, and IFN (α,β,γ), and chemokines in the amniotic sac (49, 50). There is much supporting evidence to suggest that fetal membranes do indeed respond to bacterial components and, in turn, generate cytokines (51, 52) along with many host defense peptides, which are anti-microbial in nature (53–55). During chorioamnionitis, the normal polarized distribution pattern of TLRs is completely lost, resulting in the overall upregulation of TLR2 and TLR4 expression (56).

A recent report demonstrated that human fetal membranes and neutrophils that interact directly, and LPS-stimulated factors originating from the fetal membrane, can effectively recruit, and trigger neutrophils to induce inflammatory cytokines and helps them build neutrophil extracellular traps. The effect of TLR activation in preterm infants has also been studied by checking the level of the immunomodulatory factor, such as cAMP concentration in cord blood samples along with peripheral blood samples of preterm babies for the first month after delivery (57).

### Decidua

Decidua harbors most of the immune cells, which have the capability to generate an instant immune response against invading pathogens. Immune cells, such as macrophages, dendritic cells, uterine Natural Killer (uNK) cells, and Regulatory T cells, present in decidua differentially express TLR2 and TLR4 on their surface during pregnancy (56). Additionally, resident cells in the decidua also express these pattern-recognition receptors. Transcripts of all TLRs have been found in the first and third trimester decidual cells, whereas only TLR2 and TLR4 have been found to be expressed in the first trimester decidual cells, and TLR1–TLR6 expression has been seen in the term decidua (58, 59). Furthermore, decidual cells, upon being stimulated with LPS, trigger the production of pro-inflammatory cytokines and many TLR4 pathway related downstream genes (60). These results have demonstrated the contribution of decidual stromal cells in fighting intrauterine infections and thereby act as a barrier between the developing fetus and invading microbes so as to ensure a safe environment for fetal development.

### Immune Modulation During Pregnancy

**Figure 3:**
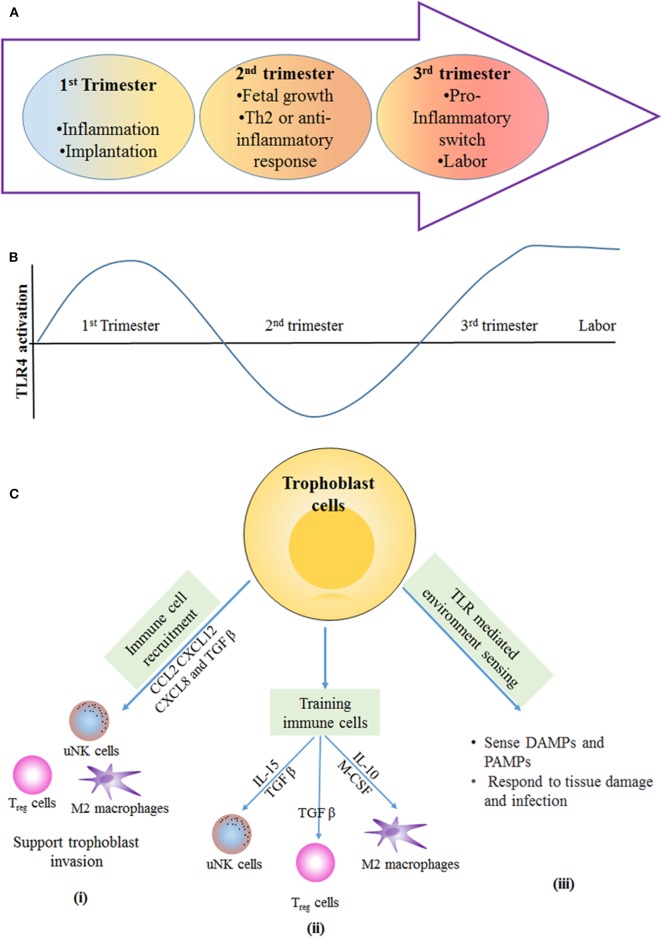
Immunological stages of pregnancy: during first trimester of pregnancy, the inflammatory response is required for blastocyst implantation. **(A)** The second trimester is described by an anti-inflammatory and T-helper 2 (Th2)-type immune microenvironment that is necessary for fetal growth. In the third trimester, switching from anti-inflammatory to an inflammatory response happens, and this is essential for labor and delivery. **(B)** Different stages of pregnancy have altered the level of TLR4 activation the first and third trimester have more TLR4 activation in immune cells and trophoblast cells, which results in inflammation that is required for blastocyst implantation and term labor and delivery. In the second trimester, the lowered TLR4 activation supports the anti-inflammatory response for fetal growth. **(C)** Trophoblast-mediated immune regulation. (i) Trophoblast cells secrete number of cytokines and chemokines, such as CC-chemokine ligand 2 (CCL2), CXC-chemokine ligand 12 (CXCL12), CXCL8, and transforming growth factor-β (TGFβ), which are responsible for recruitment of immune cells to the materno–fetal interface. Immune cells provide support for invasion and implantation of trophoblast. (ii) Trophoblast cells secrete cytokines that help in training of uterine natural killer (uNK) cells and M2-like macrophages; in turn, these immune cells support the vascular and tissue remodeling that is necessary for trophoblast invasion and differentiation. TGFβ secreted by trophoblast cells induces the polarization of regulatory T (T_reg_) cells, and these cells provide a feto-tolerant environment at the materno–fetal interface. (iii) Trophoblast cells express PRRs, such as TLRs, that allow them to sense and respond to DAMPs and PAMPs produced during tissue damage and infection. IL, interleukin; M-CSF, macrophage colony-stimulating factor.

The host graft model of pregnancy is an old paradigm that suggest that immune cells recognize the fetus as semi-allogenic and hence try to eliminate it. In the current school of thought, however, the immune cells facilitate the implantation, formation, and development of the blastocyst for the sustenance of the pregnancy. In the normal condition, there are three immunological stages: (i) the pro-inflammatory condition in the decidua that aids in implantation and placentation; (ii) the growth of the fetus occurs in an anti-inflammatory environment; and (iii) there is finally a change back to the pro-inflammatory state for parturition (25, 61, 62) (Figure 3A).

Fetus implantation in the early stages of pregnancy triggers the immune response at the junction of the decidual endometrium and extravillous trophoblast (EVT). Early contact of EVT with the maternal cells activates the immune system, primarily the innate immunity (63, 64). Innate immune cells, such as decidual macrophages, NK cells, dendritic cells, and T cells, are attracted toward the materno–fetal junction during the first trimester and remain there until parturition (65–67). These immune cells secrete different inflammatory cytokines, which are responsible for different states of the placenta. A distinct role is played by these immune cells for acceptance of the fetus and its protection from pathogens. The involvement of these immune cells and how TLR4 expression helps in pregnancy is described further (Figures 3B,C).

### Uterine NK Cells

Natural Killer (NK) cells were initially derived by their cytotoxic activity against transformed cells. These cells have a unique ability to produce cytokines and perform cytotoxic functions other than T and B cells of lymphocyte origin.

Uterine NK cells are similar to systemic NK cells, but they do not express CD16 on their surfaces. They are translocated to the endometrium lining and placenta by the chemokine secreted from trophoblast cells. Uterine NK (uNK) cells are different in that they are highly granulated and are considered to play an essential role in maintaining a successful pregnancy by cytokine production in a temporal manner (68, 69). In addition to cytokine secretion, the crosstalk of uNK cells with dendritic cells supports the production of various growth and angiogenic factors, which helps in the implantation of trophoblast toward the vicinity of maternal blood (61, 63, 65, 70). These cells are dominant until mid-gestation, which helps in the implantation and acceptance of the fetus. uNK cells do so by getting activated or inhibited by ligands expressed in invading trophoblast (HLA-C) via the KIR receptor expressed on NK cells. uNK cells help in polarizing the Th2 subset of the CD4 T-cell subsets through the activation of KIR signaling in the second stage of pregnancy. The inhibitory KIR interaction with HLA C2 (in infants or trophoblast) is associated with preeclampsia (71, 72). *In-vitro* studies have shown that uNK cells have a high TLR expression (specifically TLR 2,3, and 4), which is stimulated to produce IFN-γ or IFN-β either by TLR agonist or through other cells in the endometrium (73, 74). TLR-induced cytokines and the effector function prevents the fetus from microbial infection and provides a feto-tolerant environment. The elevated inflammatory response is balanced by IL-10 and IL-1RA, and this downregulates the pro-inflammatory cytokines (75–77). The crucial role of IL-10 was elucidated in a mouse model, which resulted in frequent PTB upon TLR4 and TLR9 activation (78, 79). It is still unclear how TLR helps in shaping the uNK population in the materno–fetal interface.

### Decidual Macrophages

In contrast to inflammatory cells, there is an abundant population of decidual macrophages, and these are critical to maintaining pregnancy after successful implantation. Decidual macrophages express CD206 and CD209 molecules on the surface along with CD11c hi/lo antigen. These cells act as antigen-presenting cells to innate (NK cells) and adaptive immune (T cells) cells at the materno–fetal interface during early pregnancy. Unlike circulating macrophages, decidual macrophages have a more M2-like phenotype and perform a “cleanup” function of apoptotic cells to prevent pro-inflammatory condition in the decidua (65, 80–83). Activation of the TLR pathway dictates the polarization of macrophages from anti-inflammatory to pro-inflammatory subsets in the uterus. Decidual macrophages have the potential to secrete cytokines like TNF-α and IL-1β along with IL-6, IL-8, and IL-10 as anti-inflammatory cytokines upon TLR agonist stimulation. TLR induced IL-10 by decidual macrophages inhibits excessive CD4 T-cell proliferation and activation (75, 84). Excessive administration of TLR ligand-like CpG or LPS modulates the macrophages to the M1 type, which leads to preterm birth or fetal reabsorption (79). Thus, the M1 phenotype of macrophages in uterus are harmful for normal pregnancy, which can be rescued either by depleting such macrophages or by administration of IL-10 cytokine (77, 85). Progesterone also prevent the NF-κB activation through TLR4 pathway in decidual macrophages, thus decreasing the production of inflammatory cytokines (86, 87).

### Regulatory T Cells

Immunology during pregnancy is similar to tumor immunology. In cancer, the adaptive immunity plays a critical role in graft rejection, but cancer cells modulate the immune cells for its establishment. As opposed to rejection, maintaining pregnancy is also a kind of allograft tolerance (61, 88). In this scenario, a subset of adaptive immunity, i.e., regulatory T cells, plays a critical role in sustaining pregnancy. Amplification of these cells helps in restraining Th1 and Th17 responses and creates an immunosuppressive environment, thus protecting the fetal allograft from elimination. T_regs_ comes into play during the second stage of pregnancy where they crosstalk with other immune cells, such as uNK, dendritic cells, and decidual macrophages, to create a “tolerant” environment by reducing the Th1 and Th17 cytokines.

The temporal existence of T_reg_ cells is regulated by TLR4 expression, which is upregulated during early pregnancy in decidual stromal cells and thus decreases the T_reg_ population. This increased TLR4 signaling inhibits the transcription factor Foxp3, which in turn reduces regulatory T-cell polarization (89). A reduced number of T_reg_ cells has been associated with preeclampsia and PTB. There is a report that, for the first time, has demonstrated the significance of regulatory T cells in a murine model, where depletion of these cells resulted in loss of pregnancy (90). Rag^−/−^ mice were treated with a TLR4 ligand (LPS), causing preterm birth; however, the adoptive transfer of T_reg_ cells rescued these mice and ensured they were able to sustain the pregnancies to term (13, 91), and negatively regulated LPS induced fetal inflammation in a late pregnancy mouse model (92). Therefore, regulatory T cells are important in maintaining a tolerant environment, and their time of polarization decides the fate of pregnancy (93). During pregnancy, a pool of memory T_reg_ cells are differentiated against the paternal alloantigen, and they are responsible for inducing tolerance upon subsequent pregnancy with the same paternal alloantigen (94, 95).

## Role of TLR4 Signaling in Pregnancy

During normal pregnancy, a large number of cytokines and chemokines are secreted by trophoblasts, which helps in the proper implantation of the embryo on the uterine wall. These cytokines also help in the training of immune cells that are essential for the establishment of different stages of pregnancy (61, 70, 96).

TLR2 and TLR4 are widely expressed on various innate immune cells, including decidual macrophages and dendritic cells. Along with these immune cells, TLR4 is reported to express in decidual cells during the first trimester, EVTs, Villous cytotrophoblasts, and hofbauer cells, though not in syncytiotrophoblasts (70). These cells protect the fetus from various microbes and infectious agents, which indicates their critical role in placenta. There are many DAMPs, such as apoptotic cells or matrix component-like fibronectin and oligosaccharides, within the placenta that trigger TLR signaling via the MyD88-NF-κB pathway. This signaling results in the production of inflammatory cytokines by neighboring immune cells in the decidua.

TLR4 expression is found in various types of cells and at different time points. Any changes in this expression or perturbation in signaling causes pregnancy disorders like preterm birth, preeclampsia, and abortion. Recently, TLR4-mediated IFN-β production and its role in pregnancy has been widely elucidated. There is an increase in the production of IFN-β by trophoblast cells upon LPS-mediated TLR4 activation via the MyD88-independent (TRIF-TBK1-IRF-3 axis) pathway. Increased IFN-β induces downstream interferon stimulating genes and also triggers negative regulators of the TAM receptor, such as Mer and Axl. Absence of these negative regulators were found to be detrimental, as fetal rejection occur in the presence of increased IFN-β in the placenta (47).

### Preterm Birth (PTB)

**Figure 4:**
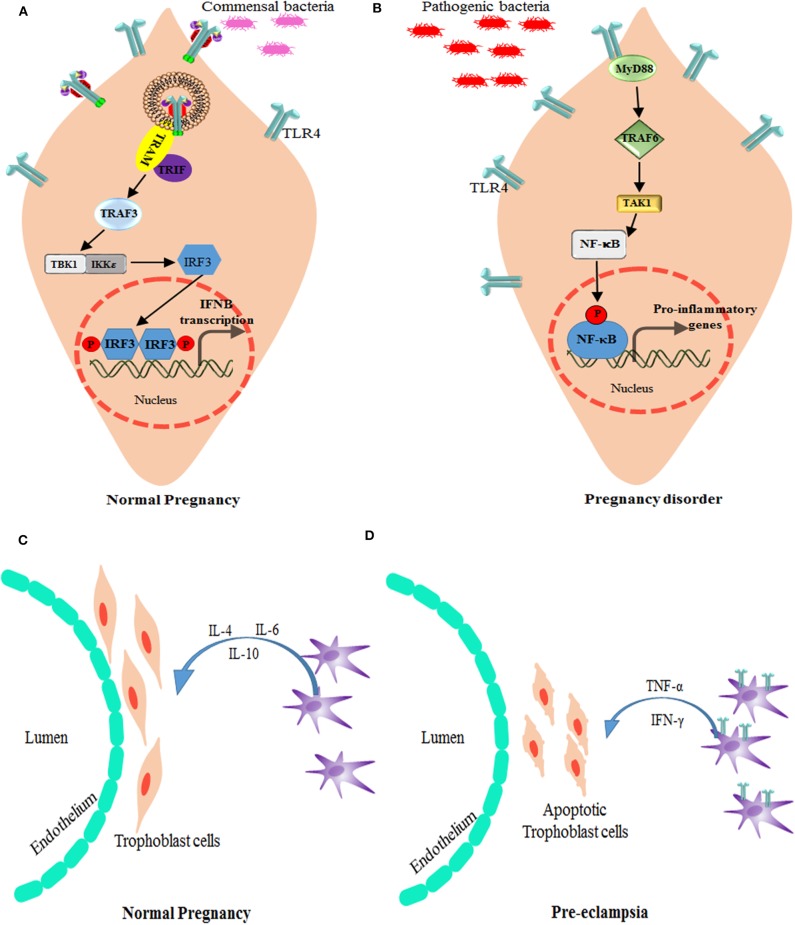
Alteration of TLR4 signaling in pregnancy disorders. **(A)** Bacterial products, such as lipopolysaccharide, activate TLR4 and the signal TRIF-TBK1-IRF-3 pathway to induce the baseline expression of IFN-β (which is encoded by *IFNB*) in the placenta. The production of IFN-β by the placenta modulates the maternal immune system and promotes tolerance, while providing protection against viral and bacterial infections. **(B)** Bacterial products stimulate the production of type I IFNs by trophoblast cells and maintain an anti-inflammatory environment as well as active surveillance and protection against infections. However, in case higher antigen load or if pathogenic infection reaches the placenta, TLR4 expression increases and modulate the activation signals through MyD88-TRAF6-NF-κB leading to inhibition of type I IFNs, and promotion of pro-inflammatory response that is responsible for preterm labor. TRAM is the TRIF-related adapter molecule (also known as TICAM2). Role of macrophages and TLR4 in Pregnancy: **(C)** In normal pregnancy, M2-like macrophages are available around spiral arteries and the endothelium, which helps in the remodeling of these arteries by producing various factors associated with angiogenesis and tissue remodeling. They also play a role in immunomodulation, for instance by producing IL-10. **(D)** During preeclampsia, increased numbers of M1-like macrophages are found in the materno–fetal interface. These M1 type decidual macrophages have more TLR4 expression and signal via NF-κB pathway to produce pro-inflammatory cytokines, such as TNF-α and IFN-γ, which induces apoptosis of the trophoblast cells.

A major problem of neonatal mortality is due to preterm labor (gestation at <37 weeks). PTB is marked by increased pro-inflammatory factors due to local or systemic infection or inflammation, such as infection in intra-amniotic (chorioamnionitis) or periodontitis, which interacts via maternal sera (97, 98) (Figures 4A,B). LPS-mediated TLR4 signaling is profound in PTB and IUFD (Intra Uterine Fetal Death) even with a low dose in LPS pre-treated mice (99). In the animal model, TLR4 knockout mice were unaffected by PTB, whereas a neutralizing antibody against TLR4 reduced fetal death in normal mice (98, 100). In chorioamnionitis, which leads to PTB, LPS-induced translocation of TLR4 toward the basal membrane is a protective mechanism to lower the immune response (101). Increased TLR4 expression on CD14^+^ monocytes has been well-correlated in patients with PTB (102). Reports suggest that small doses of LPS (TLR4 agonist) treatment in *Il-10*^−/−^ mice causes PTB, as opposed to in wild type mice (78). Also, upon LPS treatment, mice show increased uNK intrusion and placental cell death. But with depletion of uNK cells or deactivation of TNF-α, mice were rescued from PTB (103). During parturition or in preterm birth, it has been observed that TLR4 plays a critical role in developing inflammatory response by recruiting a number of monocytes and macrophages to the placenta. TLR4 and TREM-1 (triggering receptor expressed on myeloid cells 1) expression was found to be elevated in monocytes and neutrophils in patients diagnosed with PTB (104). *Tlr4*^−/−^ mice showed delayed labor due to the absence of an inflammatory cytokine storm even after LPS treatment, suggesting that TLR4 indeed is necessary for timely labor. Inflammation-induced PTB can be delayed by small molecule-like (+) naloxone, which is specific to TLR4 receptor and has the ability to cross the placenta and delay labor (105, 106). As most of the studies were done under total TLR4 knockout conditions, involvement of TLR4 activation at the materno–fetal interface was still unclear. However, in a recent study, a decidua specific conditional TLR4 knockout was generated using the *Pgr*-Cre driver (*Pgr*^Cre/+^*Tlr4*^f/f^) to explore the physiological importance of TLR4 during pregnancy. Endothelial cells expressing TLR4 has reported to be important in sensing the inflammation in the decidua, which, in turn, activates STAT3 via IL-6 in perivascular stromal cells and hence regulates the anti-inflammatory IL-10 production. The homeostasis of TLR4 expression in endothelial cells determines the pregnancy outcome, as in case of PTB, and could be a probable therapeutic target in preventing PTB (107).

### Preeclampsia (PE)

Preeclampsia is a heterogeneous disorder caused after the 20th week of pregnancy due to local or systemic abnormalities. There is much evidence to suggest that TLR signaling activation could cause PE in many ways (Figures 4C,D). An imbalance of the Th1 and Th2 response is a dominant immune response as a result of TLR4 activation which creates a pro-inflammatory environment leading to preeclampsia (108, 109). The abundance of TLR ligand could be linked to various pathogenic infection, such as *Chlamydia pneumoniae*, Cytomegalovirus, *Helicobacter pylori*, Malaria, *Toxoplasma gondii*, and Mycoplasma Hominis (110–112). Since PE is a multifactorial disorder, maternal health along with infectious load add up to the pathogenesis of this disorder. Pregnant women with urinary tract infection are also at a higher risk of this disorder (110, 113). Among all TLRs, TLR4 has been found to be associated with preeclampsia. As reported by Mazouni et al. a patient with preeclampsia showed an imbalance of the pro-inflammatory form of monocytes due to TLR2 and TLR4 signaling (114). Another factor, which is predisposed to preeclampsia, is the genetics of TLR2 and TLR4 polymorphism. Single nucleotide polymorphisms in TLR2 (Arg753Gln) and TLR4 (Asp299Gly/Thr399Ile) have been associated with early onset of preeclampsia (115), with an exception in the Caucasian population (116).

Other than different maternal syndromes, which are associated with PE, serum TLR4 and NF-κB p65 could be used as a biomarker for predicting cytokine environment and its influence on the immune cells (117). Even microRNAs (miR-155, miR-335, and miR-584), which prevents free radicals (eNOS) in the endothelial cells, are associated with PE and can be upregulated by aspirin treatment that inhibits NF-κB mediated inflammation (118).

### Placental Malaria

Parasitic infection caused by *Plasmodium* is known to stimulate various immune cells by activation of the TLR4–NF-κB axis. Placental malaria is marked by an increased innate immune response causing intra-uterine complications, decreased body weight of the fetus during birth, and susceptibility to recurrent infection in early life (119–121). The development of gestational malaria was studied in pregnant mice model infected with *P. berghei* NK65, where TLR2, TLR4, and TLR9 were identified to trigger the inflammatory pathway, leading to NF-κB activation. In this study, placental inflammation was associated with the TLR4 pathway because infection in TLR2 null and TLR9 null pregnant mice displayed no difference to that of wild-type pregnant mice. Moreover, a CD14/TLR4 blocker (IAXO-101) was successful in rescuing the malarial risk to both fetus and mother and helped in gaining the fetal body weight (122). As CD14 and lipoprotein can activate the TLR1/TLR2 pathway, inhibiting CD14 by IAXO-101 will cease the activation of TLR1/2/4 and hence affect cytokine balance, which can eventually lead to an adverse pregnancy outcome.

Under the same scenario, it was observed that the TLR4 receptor behaves differently on the maternal and fetal interface. Maternal TLR4 is involved in the pathogenesis of malaria severity, while fetal TLR4 has a protective response against placental parasite burden, which could be due to the paternal allele for *Tlr4*. Similarly, a decrease in maternal type 1 IFN receptor 1 (IFNAR1) during the course of infection promotes the parasite burden by limiting the activation and accumulation of Helper T cells. Increased fetal IFNAR1, however, helps in eliciting an anti-parasite response, but fetal IFNAR1 is not sufficient enough to reduce the placental parasite burden and its harmful effect on the fetus (6). In placental malaria, the TLR4 downstream partner MyD88 has no significant role in pregnancy outcome irrespective of maternal or fetal genetic background when infected with *P. berghei* NK65. The deletion mutant of MyD88 did not produce any abnormalities and affected growth in infected pregnant mice (123). An ideal vaccine approach against TLR4 could be formulated that can be specific to placental malaria and would provide protection against maternal anemia, PTB, and fetal growth retardation.

## Therapeutic Modalities for Pregnancy Related Disorder Targeting TLR4 Signaling

Various TLR4 antagonist and inhibitors have been developed that are currently in different phases of clinical trial for diseases other than pregnancy. There are few options that are currently being studied for immune modulation and inhibition of TLR expression for pregnancy-related disorders. The association of TLR4 was studied in women with aPL (antiphospholipid antibodies), which activate the TLR4 pathway and the inflammatory response in trophoblasts leading to miscarriages, PE, and PTB (124). Recent studies have identified endothelial TLR4 to be a potential therapeutic target for PTB (107). Cytokines like IL-6 have been successful in delaying preterm birth by immunomodulation and regulating prostaglandin-related genes (125).

**Figure 5:**
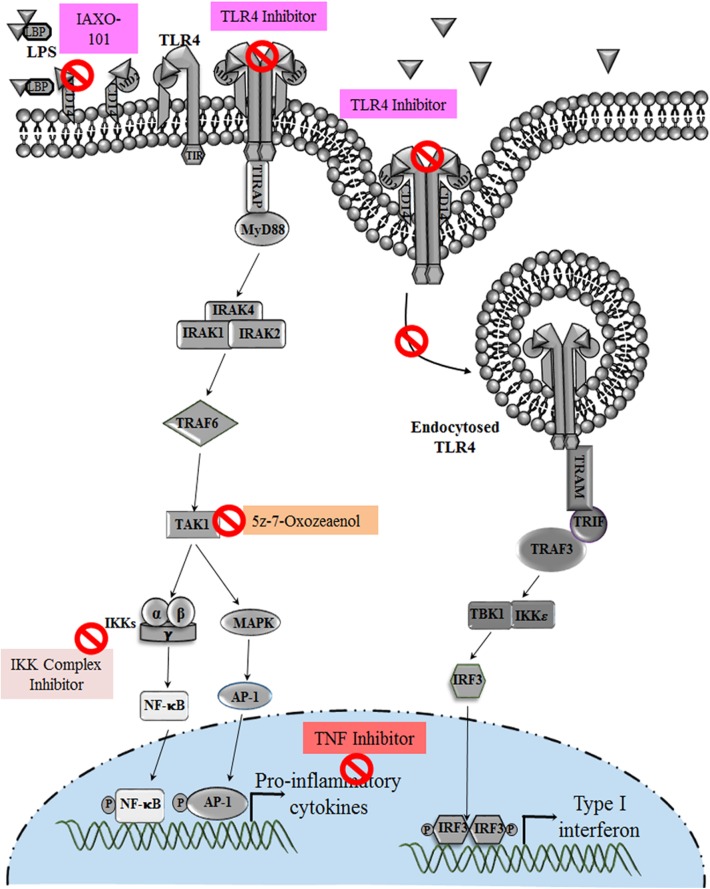
Various Drugs that target TLR4 pathway in pregnancy disorders: drugs and anti-inflammatory agents that target TLR4 pathway and its downstream molecules during infection induced preterm birth. Hormones and drugs targeting TLR4 expression help in switching the pro-inflammatory environment to anti-inflammatory in various pregnancy disorders. TNF inhibitors reduce the increased TNF production during altered TLR4 activation in preeclampsia.

Cytokine-suppressive anti-inflammatory drugs (CSAID's) are a novel group that target the NF-κB and MAP Kinase pathways, making them more effective than Non-Steroidal Anti-inflammatory Drugs (NSAID). CSAIDs that can selectively inhibit TAK1 and the IKK complex are well-studied in animal models, which has resulted in the reduction of cytokines and prostaglandin levels (126) (Table 1). TAK1 inhibitor 5z-7-oxozeaenol (OxZnl), a resorcyclic acid lactone that is an excellent pharmacological target in CSAIDs, can effectively block the cytokine cascade to avoid preterm birth (143, 144) (Figure 5). Although these drugs can selectively target TLR-NF-κB pathway, there are some side effects associated with its use, such as how it may inhibit unwanted NF-κB activation, thus increasing the predisposition to opportunistic infection. To resolve such problems, these drugs can be administered in amniotic cavity to reduce the side effects and enhancing the efficacy of the drug. But the probable benefits and the risk assessment should be balanced, and such CSAID therapy should be given to women who can gain significant benefits.

**Table 1:** List of drugs targeting TLR4, and its downstream signaling molecules during pregnancy disorder.

**Drugs**	**Role in pregnancy disorder**	**Disorder**	**References**
**TNF-α** **ANTAGONIST**			
Hydroxyquinone	Reduces production of TNF and endothelin-1	PE	(127)
Asprin	Prevents endothelial dysfunction due to TNF	PE	(118)
**TLR4 INHIBITOR**			
Curcumin	Downregulates TLR4 expression and NF-κB mediated inflammatory response	PE	(108, 128)
Vitamin D	Calcitriol can modulate innate as well as adaptive response (pro to anti- inflammatory) Decreases TLR4 expression	PTB, PE & spontaneous miscarriages PE	(129, 130) (131, 132)
Rosiglitazone	Reduces TLR4 mediated inflammation Increases antioxidant response by NRF-2 and HO-1	PTB	(133)
Progesterone	Inhibit TLR4 expression in macrophages Promotes Th2 differentiation Induces tolerance at materno–fetal junction	PE	(86, 134) (135) (136, 137)
**IMMUNOMODULATORS**			
*Inonotus obliquus* polysaccharide	Maintain Th17/T_reg_ cell balance	Infection of *T.gondii*	(138)
IL-10	Maintains anti-inflammatory condition in decidua	PTB	(107)
**IKK COMPLEX INHIBITOR**			
NEMO-binding Domain Inhibitor	Reduces Prostaglandin E2 (PGE2) in LPS and *Ureaplasma parvum* stimulated *in-vitro* ovine gestational membrane model	PTB	(139)
Parthenolide	Reduces inflammatory gene expression in patient derived choriodecidual cells. Decreases TNF-α and COX-2 expression in human urothelial cell stimulated with TNF-α.	PTB	(140, 141)
TPCA-1	Similar effect as of parthenolide. Reduction in PGE2 level in LPS stimulated ovine pregnancy model	PTB	(139, 140, 142)

## Concluding Remarks

Detailed study of spatiotemporal expression of TLRs during normal pregnancy and related disorders using various model systems has increased our understanding of placental infections and furthered our development of strategies to overcome the adverse pregnancy outcomes. Activation of innate immune PRR through TLR4 at the materno–fetal interface ensures that the developing fetus is protected from invading pathogens at early stage of pregnancy. But uncontrolled activation of TLR4 has been proven to trigger chronic inflammation and to result in loss of pregnancy. Hence, increased levels of TLR4 on leucocytes or cells of maternal and fetal origin could be used as a biomarker for pregnancy disorders. Many studies have shown the involvement of innate immune cells for sustaining a successful pregnancy.

It is not yet clear how the TLR4 expression pattern alters during various stages of pregnancy and in what way its uncontrolled activation on immune or other decidual cells at the maternal–fetal interface leads to various pregnancy failures. Addressing this issue may help in developing certain clinical diagnostic markers as well as specific antagonists targeting either TLR4 specifically or its downstream effector molecules for improving pregnancy outcomes.

## Author Contributions

PF and VS contributed equally in writing the review. Conception of Idea was done by SC, PF, and VS. Manuscript writing and editing was done by all the authors.

## Conflict of Interest

The authors declare that the research was conducted in the absence of any commercial or financial relationships that could be construed as a potential conflict of interest.
